# Picture Norms for Chinese Preschool Children: Name Agreement, Familiarity, and Visual Complexity

**DOI:** 10.1371/journal.pone.0090450

**Published:** 2014-03-05

**Authors:** Lamei Wang, Chia-Wen Chen, Liqi Zhu

**Affiliations:** 1 Key Lab of Behavioral Science, Institute of Psychology, Chinese Academy of Sciences, Beijing, China; 2 Assessment Research Center, The Hong Kong Institute of Education, Hong Kong, China; University of Hertfordshire, United Kingdom

## Abstract

Pictorial stimuli standardized for Chinese children are still absent although it is needed in order to test the development of children's cognitive functions. This study presents normative measures for Snodgrass and Vanderwart pictures, viewed by 4- and 6-year old Chinese children. Name agreement, familiarity, and visual complexity were obtained for each age group. The data indicate substantial differences between young and older children in name agreement based on expected name, familiarity and visual complexity. The correlation pattern of the variables collected in the present study were consistent with children's norms in other languages and norms of Chinese adults, while there are cross-age and cross-culture differences in specific variables. The obtained measures represent a useful tool for further research on Chinese children's pictorial processing and constitute the first picture normative study for children in this language.

## Introduction

Picture norms are necessary when psychologists want to employ pictures to investigate the development of cognitive functions. Pictures of everyday objects are more often used in both semantic and episodic memory tasks [1]. Snodgrass and Vanderwart [2] first introduced a standardized set of 260 pictures, which consisted of black-and-white drawings of objects. Their norms of name agreement, familiarity, and visual complexity for American adults have since been widely used in psychological and neuropsychological studies. For example, such pictures have been used in studies that: compare naming and recognition between normal and brain-damaged subjects [3]; identify neural correlates of category-specific knowledge [4]; and investigate the semantic processing of words and pictures [5]. By using the same normative stimulus material, these studies enable more direct inter-study comparisons. However, because the properties participants ascribe to pictures, such as names, familiarity and complexity differ across cultures and ages [6], [7], [8], [9], such norms should be collected for different cultures and age groups.

In the past two decades, a great many studies have provided cross-cultural validation for Snodgrass and Vanderwart's [2] pictures in different languages. Normative data for them have been re-examined and provided using other young American adult samples [7], [10]. Examples of their norms in different languages include Brazilian [11], Dutch [12], [13], French [14], [15], Greek [16], Icelandic [17], Italian [18], Japanese [19], Portuguese [20], Russian [21], Spanish [22], [23], and also comparisons spanning seven languages [6].

As far as Chinese is concerned, Shu, Cheng, and Zhang [24] reported normative data on rated concept familiarity for 232 of Snodgrass and Vanderwart's [2] pictures, as well as measures of name agreement, image agreement, and visual complexity in Chinese adults. Weekes, Shu, Hao, Liu and Tan [25] added rated age of acquisition (AoA) to Shu et al.'s [24] norm and then examined the predictors of timed picture naming in Chinese adults. Yoon et al. [9] presented normative measures of name agreement, concept agreement and familiarity for Snodgrass and Vanderwart [2] in younger and older Chinese and American adults. Liu, Hao, Li and Shu [26] offered timed picture naming norms of 435 line drawings which included 200 pictures from Snodgrass and Vanderwart [2] for Chinese adults. However, no Chinese children's picture norm has been developed so far.

Following these norms collected for Chinese adults, we chose Snodgrass and Vanderwart [2] pictures for the collection of name agreement, familiarity, and visual complexity for Chinese children in the current study. Though there were some proposed disadvantages of this picture set in its lack of color information [27] and its imparity in recognizing line-drawings of living and non-living things [28], Snodgrass and Vanderwart's [2] is the first and most widely used picture set with norms in many different languages including Chinese (adults), and especially with norms for American and French children, collecting Chinese children norms for it makes cross-age and cross-culture comparison possible.

Among all the above picture norms from different cultures, only a few focused on children. This is surprising as pictorial stimuli are very often used to test children, given that pictures are more age-appropriate than words for pre-reading children. There has been evidence that name agreement, familiarity, and visual complexity differ between young children and adults [29], [30] with the children's rating lower almost on all measures. Therefore picture norms for children are required when pictures are employed to explore children's memory or other cognitive functions. Berman, Friedman, Hamberger, and Snodgrass [29] developed picture norms of name agreement, familiarity, and visual complexity for 7- to 10-year old American children with Snodgrass and Vanderwart [2] pictures. Cycowicz, Friedman, Rothstein, and Snodgrass [30] provided additional data of 400 pictures for 5- and 6-year old American children. Pompéia, Miranda and Bueno [31] provided normative measures for the same 400 picture objects [30] for Portuguese speaking Brazilian 5- to 7-year old children. Furthermore, Cannard, Blaye, Scheuner and Bonthoux [32] have examined picture name agreement in 3- to 8-year old French children, using a set of 145 Snodgrass and Vanderwart [2] pictures, which they considered to be appropriate for French children. However, to our knowledge, so far there are no available standardized picture norms for Chinese children. All the Chinese norms mentioned before had been done for Chinese adults. Therefore, setting up systematic picture norms for Chinese children becomes necessary.

To build such norms, first we need to define the suitable age for our data collection according to the picture norms of children collected in other cultures. Berman et al. [29] found that the differences of ratings between 7- to 10-year old American children and adults were trivial for most pictures, thus concluding that judgments of familiarity, complexity, and the names of line drawings of common objects are based primarily on information processing accomplished prior to age 7. Cycowicz et al. [30] indicated that 5- to 6-year old American children's name agreement, and ratings of familiarity differed widely with older children and adults with young children rating lower on both measures. Pompéia et al. [31] also indicated that 5- to 7-year old children's name agreement and ratings of familiarity and complexity differed from adults'. According to D’Amico et al. [33], 5- and 6-year old Italian children were much slower and less consistent than adults in producing the target name. Cannard et al. [32] demonstrated that among 3- to 8-year old French children, the 3- and 4-year old children produced many more alternative answers than the 6- to 8-year olds. From the age of 5 onward, the percentage of different first names decreased. All these findings indicate that preschool years (3 to 6 years of age) show the most differences with adults in all measures. Furthermore, given that preschool children are at the pre-reading stage, pictorial stimuli are necessary materials instead of words to test their cognitive functions. Therefore, we took preschool years as our target age groups; and in order to illustrate potential age differences, we involved K1 (kindergarten first year, around 4 years old) and K3 (kindergarten third year, around 6 years old) children who were two years apart in age on average.

Children's picture naming performances have been proposed to be influenced by different picture attributes such as visual complexity [29], [30], [33] and age of acquisition [30]. Age of acquisition has also been suggested to influence adults' naming latency [34] and naming accuracy of patients with probable Alzheimer's disease [35]. Furthermore, it has been proposed that familiarity is a strong predictor of adults' picture naming [14], [34] while visual complexity affects the memory ability for the pictured object [14]. Following picture norms for children in other cultures (for example, [30]), our normative data will include the most common name given to each of the 260 concepts (modal name), name agreement, picture familiarity, visual complexity, and word length. In addition, we present the alternative names produced by our sample of children. Word frequency measures of 246 expected names were taken from Cai and Brysbaert [36] and objective AoA of 221 pictures were taken from Liu et al. [26] based on 442 children's rating.

Name agreement is an essential predictor of naming difficulties during the process of picture naming, thus it is important in investigating recall, recognition and verbal coding [31]. Naming process models argued that participants first initiate visual recognition processes and then activate semantic information, prior to lexical selection and phonological encoding [37]. Therefore the picture's attributes, the possible names the picture can have, and the participants' knowledge of pictures' names can influence picture-naming performance [32]. To provide standardized picture norms, it is necessary to determine a picture's most common name and the degree of name agreement between participants [18], [30], [32].

There have been three different methods on the name agreement measurement. The first is the classic H statistic, which offers information for the variability of responses in a group of participants [2]. It is calculated from all the alternative answers but does not take into account “do not know name” (DKN) or “do not know object” (DKO) responses, which nevertheless is common especially among children. Furthermore, the computed H value is based on the modal name, but the modal names produced by children do not necessarily correspond to adults' modal names. The second measure, percentage of agreement based on model names [2], corresponds to the percentage of participants naming the picture based on the modal names. According to Cannard et al. [32], it is not a good indicator of correct picture naming in the population of children either, for the same reason that the modal names produced by children might be different to adults' modal names. The third measure is the percentage of agreement based on the expected name [32], which is calculated by taking the proportion of all responses (i.e., including all DKNs and DKOs) to a picture representing the expected name response within each group. It has been demonstrated to be a better measure of picture-naming performance, especially when the subjects are children, because young children are more variable than adults in their naming responses and less likely to know the object or its name [32]. In the current study we will adopt percentage of agreement based on the expected name as the measurement for name agreement. The information statistic H was also provided in order to make comparison with other norms.

The present study aims to create a normative database for pictorial materials that will be useful in a wide range of cognitive experiments with Chinese preschool children. This goal has been achieved by providing the following normative data of pictorial stimuli for Chinese children.

## Methods

### Participants

Sixty-six children in kindergarten first year (K1, 32 kids, half girls, *M* = 4.11, *SD* = 0.45) and third year (K3, 34 kids, half girls, *M* = 5.95, *SD* = 0.64) participated in the study. All children were native Chinese Mandarin speakers, and their language abilities are typically developing as assessed using the Verbal Index of Revised Chinese version of the McCarthy Scales of Children's Abilities (MSCA-CR) (*M* = 54.49; *SD* = 9.88). Written informed consent from the next of kin, caretakers, or guardians on the behalf of the children participants were obtained. After task completion subjects received small gifts. The protocol was approved by the Institutional Review Board (IRB) at the Institute of Psychology, Chinese Academy of Sciences.

### Materials

The pictures were unambiguous line drawings of 260 common objects taken from the adult norms of Snodgrass and Vanderwart [2]. The 260 pictures were randomly divided into four blocks, each containing 65 items. The order of blocks was counterbalanced across subjects. Each block was shown with the E-prime program in random order. Every subject watched all 260 pictures on a 15-inch Lenovo laptop.

### Procedures

Each child was tested individually at the kindergarten he/she attended during a procedure of four sessions (one block per session), each of which took from 20 to 30 minutes to complete. Order of the sessions was counterbalanced across subjects. The child viewed one picture at a time on the laptop computer screen. The picture remained on the screen until the child provided the experimenter with information about the name, familiarity, and visual complexity of the picture. Children were given at least five minutes of rest between sessions. Some children were tested two sessions each day in two continuous days according to his/her state of attention. (Results obtained from children tested on two different days showed no significant differences from the results of children who evaluated all pictures on the same day. And there was no order effect with the results.) Instructions were adapted from those published by Cycowicz et al. [30]. A short practice block of four line-drawing pictures of similar style taken from another picture set [29] was presented before the formal procedure.

For the naming task, children were told to give the first name that came to mind, and that a name could consist of more than one word. When a child could not name the picture, questions were asked that would aid in determining whether the child did or did not know the concept. This is important because young children comprehend many concepts that they fail to express verbally. The experimenter asked questions like ‘‘what can you do with it?’’ or ‘‘Where do you see it?’’ If the answer indicated that the child did have knowledge about the object, then the naming was recorded as “DKN” (don’t know name) and he/she was asked to answer the familiarity and the visual complexity questions. For cases in which the child did not recognize the object depicted at all, the naming was recorded as “DKO” (don’t know object) and the next picture was presented.

We followed Cycowicz et al. [30] to score children's ratings of familiarity and visual complexity on a three-point scale rather than a five-point scale, as Berman et al. [29] had suggested that even older children (ages 8–10) could not assign ratings across the full range of numerical values in the five-point rating scale. All the previous picture norms collected for children [29], [30], [31], [32] adopted this three-point scale. And our pilot study has also confirmed that the three-point rating scale was more suitable for preschool children.

For the familiarity rating, the child was asked ‘‘how often do you see or think about this object?” (“Ni jing chang kan dao huo xiang dao ta ma?” in Chinese). Answer choices ranged from ‘a lot’ (scored 5) to ‘sometimes (scored 3) to ‘very little’ (scored 1), as in Cycowicz et al. [30]. To help the children grasp the scale, in the practice block, children were shown a picture of a dog and told that if they have a pet dog, they should say “a lot,” even though the picture may not look much like their own dog, because the question refers to how much they see or think about any dog. To anchor their judgments at the other extreme, in the practice block, the “harp” slide was shown as an example of an object most people do not see or think about very often. However, the children were told that if someone they know has that object, they might want to choose one of the answers “a lot” or “sometimes” according to the frequency of seeing it. To reduce the effects of response bias, the children were encouraged not to rate all pictures using the same one or two points on the scale, but, rather, to make sure the most familiar concepts were rated “a lot,” the least familiar “very little,” and others “some.”

For the visual complexity rating the child was asked “How difficult is it to draw or to trace this picture?” (“yao hua ta huo zhe miao ta nan ma?” in Chinese). Answer choices ranged from ‘hard’ (scored 5) to ‘medium’ (scored 3) to ‘easy’ (scored 1). The children were told that if the picture contains many small lines, they should answer “hard,” even if they are very good at drawing or tracing. They were told to pick “easy” if the picture had very few lines, even if they did not like to draw. The “bee” and “balloon” pictures were shown in the practice block as examples of hard and easy pictures, respectively.

We asked children to rate the image agreement (IA) in our pilot study. Following Snodgrass and Vanderwart's [2] procedure of rating IA, we required children to form a mental image of the object named, then to rate the degree of agreement between their image and the picture. However, due to the very young age of children in our study, it was difficult to let them understand how to form a mental image and then compare it with the real picture. They answered ‘high agreement’ almost to all pictures, thus we excluded this measure in the formal study.

### Dataset

The following information was obtained for each picture of objects.

#### Expected name

The expected name corresponds to the most frequent name given in a language, in this case Chinese, for a pictured object. Among all the available picture naming norms for Chinese adults, only Yoon et al. [9] provided norms of the full set of 260 Snodgrass and Vanderwart [2] pictures, so we took it as a main reference. Considering the possible effect of social change on naming, we compared it with the most recent model names provided by Chinese adults in Liu et al. [26] while checking the intended concept in Snodgrass and Vanderwart [2] when there are obvious inconsistencies. In total we replaced 10 modal names from Yoon et al. [9] with the more appropriate modal names from Liu et al. [26] as expected names (see Table S1).

#### Modal name

A modal name is defined as the name given by the majority of subjects. In order to compare modal names with the corresponding expected names, the scheme provided by Snodgrass and Vanderwart [2] was followed, in which these names were classified into synonyms, superordinates, subordinates, components, coordinates, and failures. The first 5 categories are self-explanatory, and “failures” were defined as names given that either were visually similar to or had no relationship to the expected names. “Failure” responses included names that were non-nouns (e.g., 修东西 [to fix stuff]), non-object (e.g., 音乐 [music]) or did not accurately describe the concept. Two trained judges (university students) classified the modal names that were different from expected names into one of the above categories. A third judge (L. W.) resolved discrepancies when the judges did not agree with each other. The inter-rater agreement prior to the third judge was 85.09% (for 137 of the total of 161 modal names that were different from expected names).

#### Word length

The number of Chinese characters in the modal name constituted word length.

#### Name agreement

We offer two measures of name agreement. The first is the information statistic, H, which is computed for each picture by the formula (taken from Snodgrass & Vanderwart [2])
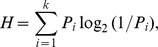
where k refers to the number of different names given to each picture and P_i_ is the proportion of participants who gave each name. The computation of H does not take into account “do not know name” (DKN) or “do not know object” (DKO) responses (for more information see [2]). For the H measure, a lower number signifies greater name agreement. For example, when all subjects supply the same name, the value is zero. A higher value indicates that a greater number of alternative names were supplied.

The second measure of name agreement is the “percentage of agreement based on the expected name.” It was demonstrated to better represent the meaning of name agreement than the two classical measures (the percentage of agreement based on modal name, and the H statistic based on alternative names), as there are high rates of no responses in young children [32]. Percentage of agreement based on the expected name was calculated by taking the proportion of all responses to a picture representing the expected name response within each group. A higher number signifies greater name agreement.

#### DKN and DKO

Number of responses on “don’t know name” and “don’t know object,” which has been described in the Procedures section.

#### Alternative Names

The criteria used for counting different instances of names were a combination of those used in Snodgrass and Vanderwart [2] and Yoon et al. [9]. First, all name responses were recorded, with any wrongly written characters (e.g., homonyms or errors) corrected. Second, any articles, quantifiers and unnecessary adjectives accompanying name responses were not retained (e.g., 一个 [a], 小 [small], 大 [big], 漂亮的 [beautiful], 爷爷的 [grandpa's]). Finally, as in Yoon et al. [9], a special criterion as following was imposed to accommodate conventions specific to the Chinese language. In Chinese, certain responses are deemed identical in their colloquial usage (e.g., 钟表 and 钟 [clock]; 电视 and 电视机 [television], 水井 and 井 [well]; 盒子 and 盒 [box]). All such responses were merged where appropriate and thereafter counted as the same. In addition, any elaborations (e.g., 食指 [index finger] and 手指 [finger]) were counted as separate name responses.

#### Familiarity

As noted above, participants used a 3-point (scored as 1, 3, 5) rating scale to indicate their degree of familiarity with each object, as in other children picture norms [29], [30], [31], [32]. When a participant did not know the object depicted (DKO), a familiarity rating was not generated. Such occurrences were therefore not included in computing means (i.e., only the actual number of subjects who supplied ratings was used in computing mean values).

#### Visual complexity

It was also indicated in a 3-point (scored as 1, 3, 5) rating scale. When subjects did not know the object depicted (DKO), a rating of visual complexity was unavailable. As for familiarity, such occurrences were not included in computing the means.

#### Word frequency

We took the word frequencies of 246 expected names from Cai and Brysbaert [36], which is a reliable source of Chinese word frequency measures recently published. They assembled a database of word frequencies based on a corpus of film and television subtitles (33.5 million words). Word frequency was presented by the log10 of the number of films in which the word was observed.

#### Age of acquisition (AoA)

The objective ages of acquisition of 221 of the 260 Snodgrass and Vanderwart [2] concepts were taken from Liu et al. [26], which has taken a big sample of children to collect the objective age of acquisition.

## Results

The data of young children and older children were analyzed separately. Independent samples t-tests were used to compare the data of the young children with the data of older children. Then we conducted Pearson correlation analysis to explore the relationship between the various variables. Finally, we compared our norm with Chinese adults from Liu et al. [26], American and French norms for children to identify cross-age and cross-cultural correlations and differences.

Appendix S1 presents the norm data of Chinese young children (K1) and older children (K3) for the 260 pictorial stimuli, listed in alphabetical order according to their presentation in Snodgrass and Vanderwart [2] (Items 1–260). The norm includes Expected Name, Modal Name of each age group, Length of modal name, the English translation of modal name, Name Agreement (H, and % based on Expected name), Number of DKN, DKO and Alternative Names, Familiarity, and Visual Complexity. It also includes the summary statistics for Word Frequency from Cai and Brysbaert [36] and Objective AoA (age of acquisition) reported by Liu et al. [26]. Table S2 contains the summary statistics for the measures obtained for Chinese young children (K1) and older children (K3). The detailed results of all measures are listed as following.

### Modal Name

After excluding the DK responses (DKN & DKO), the minimum and maximum numbers of responses for each picture are 14 and 32 in K1 children, and are 19 and 34 in K3 children respectively. The children from K1 provided modal names that differed from the expected names for 88 of the 260 stimuli (33.85%). More than half of the cases (46) were classified as superordinate; for example, young children gave name 衣服 [clothes] to six items, including blouse, jacket, coat, shirt, sweater and vest. Eight concepts were classified as synonyms, for example, 盒子 [box] for 箱子 [box]. Fifteen concepts were classified as Failures, for example, 车 [car] for 炮 [cannon]. Coordinate names occurred 15 times and included naming substitutions such as 白菜 [cabbage] for 芹菜 [celery] and 苹果 [apple] for 桃子 [peach]. Two modal names given were component names of the concept such as 线 [thread] for 线轴 [spool of thread]. The remaining two names were subordinate, such as 红旗 [red flag] for 旗[flag].

The children from K3 provided modal names that differed from the expected names for 73 of the 260 stimuli (28.08%). Thirty-two concepts were classified as superordinate, for example, 琴[musical instrument] for 手风琴 [accordion] and 竖琴 [harp]. Coordinate names occurred 13 times and included naming substitutions such as 萝卜 [radish] for 胡萝卜 [carrot]. Twelve concepts were classified as synonyms, for example, 蚂蚱 [locust] for 蝗虫 [locust]. Ten concepts such as 盒子 [box] for 面包机 [toaster] were classified as Failures. Four modal names given were component names of the concept such as 脚 [foot] for 腿 [leg]. Two concepts were classified as subordinate; for example, 公鸡 [cock] for 鸡 [chicken]. The numbers of the modal names classified into different categories are shown in Table S3.

There was a tendency for younger children to provide more superordinate names than older children as you can see from Table S3. Two concepts were classified as Failures among both age groups. These are “thimble” and “nail file.” In the case of the thimble, the majority of the children called it 垃圾桶 [garbage can], due to its visual similarity. The picture of the nail file was recognized by most children as a 刀 [knife], a visually similar object.

There are 14 items which none of the subjects could name with the expected names, including 锚 [anchor] (Item 4), 棒球捧 [baseball bat] (Item 19), 骆驼 [camel] (Item 43), 雪茄 [cigar] (Item 58), 香烟 [cigarette] (Item 59), 长笛 [flute] (Item 92), 圆号 [French horn] (Item 99), 烫衣板 [ironing board] (Item 124), 锉刀 [file] (Item 152), 电插头 [electric plug] (Item 177), 水舀子 [water ladle] (Item 179), 唱机 [record player] (Item 184), 灯罩 [lampshade] (Item 230) and 脚趾 [toes] (Item 235). Another 6 items were named as the expected names by all the subjects, including 大象 [elephant] (Item 84), 脚 [foot] (Item 94), 眼镜 [eyeglasses] (Item 105), 蘑菇 [mushroom] (Item 150), 袜子 [sock] (Item 211), and 太阳 [sun] (Item 222).

### Name agreement

The measure of name agreement expressed by the information statistic H showed no significant differences between the young children and the older children in the independent-samples t-test (*t* (518) = .55, *p* = .582) based on the items (the t-tests hereafter were all based on items). However, the percentage measure of name agreement showed significant difference between them (*t* (518) = −2.542, *p*<.05, Cohen's D = −.222), with the older children (*M* = .56, *SD* = 0.35) higher than the young children (*M* = .48, *SD* = 0.36).

### DKN and DKO

The maximum number of DKN is 11 in K1 children regarding to two items 门把手[door knob] (Item 77) and 熨斗 [iron] (Item 123), and is 9 in K3 children regarding to the item 唱片机 [record player] (Item 184). The maximum number of DKO is 12 in K1 children regarding to the item 纺车 [spinning wheel] (Item 213), and is 7 regarding to two items 改锥 [awl] (Item 56) and 纺车 [spinning wheel] (Item 213). Put together, the maximum number of DK responses (DKN & DKO) is 18 in K1 children regarding to the item 改锥 [awl] (Item 56), and is 15 in K3 children regarding to the item 纺车 [spinning wheel] (Item 213). The number of DKN ((*t* (518) = 3.669, *p* <.01, Cohen's D = 0.322) and DKO (*t* (518) = 5.341, *p*<.001, Cohen's D = 0.469) of the two age groups both revealed significant differences. This indicates that the failure number of naming (K1, *M* = 12.147, *SD* = 13.614; K3, *M* = 7.559, *SD* = 11.173) and recognizing pictured objects (K1, *M* = 28.088, *SD* = 60.139; K3, *M* = 6.118, *SD* = 12.941) is higher among younger children compared to older ones.

### Alternative Names

Alternative names given for each picture by each age group are presented in Appendix S2. Both young and older children are variable in their naming responses, and many of the concepts were assigned a large number of alternative names. The number of Alternative Names for the two age groups were not significant, *t* (518) = −0.749, *p* = 0.454. Young children (*M* = 4.25, *SD* = 3.29) gave similar number of alternative names with older children (*M* = 4.49, *SD* = 3.94). Cannard et al. [32] demonstrated that among 3- to 8-year old French children, the 3- and 4-year old children produced many more alternative answers than the 6- to 8-year olds. Perhaps the age range in the current study (4- to 6- year olds) was not large enough to show a significant difference in number of alternative names.

### Familiarity

The difference of familiarity between young children and older children was significant (*t* (518) = −5.254, *p*<.001, Cohen's D = −0.462). Familiarity was lower in young children (*M* = 3.59, *SD* = 0.69) than in the older children (*M* = 3.88, *SD* = 0.51).

### Visual complexity

The difference of visual complexity between young children and older children was also significant (*t* (518) = 9.923, *p*<.001, Cohen's D = 0.872). Visual complexity was larger for young children (*M* = 2.70, *SD* = 0.40) than for the older children (*M* = 2.31, *SD* = 0.49).

### Correlation Matrix

Each of the variables measured plays an important role in various cognitive tasks. It is necessary to know the relationships among the measures. Table S4 shows the correlation matrix of different variables with combined data of two age groups. The significant correlation coefficients were marked with asterisks.

The two measures of name agreement (H and %) show a high negative correlation as it should be. For the percentage measure, a higher number signifies greater name agreement. However, for the H measure, a lower number signifies greater name agreement. Thus the significant correlations between each of two name agreement measures (H and %) with other variables are always in opposite direction. Variables of Familiarity, Visual Complexity, Frequency and AoA are all correlated with both name agreement measures. The correlations between AoA and name agreement measures indicate that for concepts acquired at an early age the level of name agreement is high. Children's name agreement measures correlate less with word frequency than with AoA, which is similar to the finding of Cycowicz et al. [30].

### Comparison with Chinese adults' norm

Correlation between Chinese K1 and K3 children in the current study with Chinese adults (data of 221 Snodgrass and Vanderwart pictures from Liu et al. [26]) revealed relatively high correlations overall for name agreement H, familiarity, and visual complexity (see Table S5). The correlation pattern of the variables collected in the present study was consistent with normative studies in Chinese adults. Nevertheless, the comparison between preschool children and adults revealed significant differences in picture familiarity (*t* (220)  = 18.99, *p*<.001, Cohen's D  = 2.56). Preschool children rated lower familiarity (M  = 3.75, SD  = 0.56) than adults (M  = 4.52, SD  = 0.60). No statistically significant difference was found in other variables.

### Comparison with American and French children's norms

To compare the present norm with American and French children norms established by Cycowicz et al. [30] and Cannard et al. [32], we show correlation coefficients between the present norm and those obtained for the same set of pictures in Table S6. The coefficients demonstrated mediate positive correlations, ranging from.371 to.830, and they were all significantly different from zero. And the correlation patterns of the variables were similar between children norms in different cultures.

However, significant differences were found between Chinese and American children in familiarity (*t* (259) = 28.97, *p*<.001, Cohen's *D* = 3.60), complexity (*t* (259) = −13.32, *p*<.001, Cohen's *D* = 1.65) and H statistic (*t* (259) = 10.73, *p*<.001, Cohen's *D* = 1.32). Chinese children had higher familiarity (*M* = 3.73, *SD* = 0.57) and H statistic (*M* = 1.52, *SD* = 1.08) than American children (familiarity, *M* = 2.67, *SD* = 0.46; H, *M* = 0.88, *SD* = 0.78), but lower complexity (*M* = 2.62, *SD* = 0.44) than American children (*M* = 3.04, *SD* = 0.81). Compared to French 4- and 6- years old children, Chinese children had significant lower (K1: Chinese, *M* = 57.43, *SD* = 35.13; French, *M* = 66.97, *SD* = 30.88; K3: Chinese, *M* = 64.13, *SD* = 32.69; French, *M* = 83.40, *SD* = 23.92) Agreement based on Expected Name (K1, *t* (142) = 3.46, *p*<.001, Cohen's *D* = 0.58; K3, *t* (142) = 7.09, *p*<.001, Cohen's *D* = 1.19).

## Discussion

The present study is the very first to provide a standardized picture norm for Chinese preschool children. The properties of the classical Snodgrass and Vanderwart [2] pictorial stimuli, which have been used very extensively in neuropsychological and psycholinguistic literature across many different cultures, have been provided and quantified for Chinese children. Norms of modal name, word length in characters, the percentage of name agreement, H value, familiarity, and visual complexity were made available for Chinese 4- and 6- year old preschool children separately. Age-related differences of these variables between the two age groups were also explored.

Young children were less accurate than older children in naming the pictures, which is reflected in lower name agreement measure based on expected names. Given that K1 and K3 children are on average two years apart, and this is a time frame where the older children should be acquiring a great deal of knowledge and associations of the world, there should be differences in naming ability between these two age groups. However, such differences were only illustrated by “percentage based on expected names” but not by H measure. This result confirms the hypothesis that “percentage based on expected name” is a better measure than H measure. We explain this by the large percentage of DK responses included in calculating the H value.

The familiarity level of the pictures for young children was lower than that for the older children, which are consistent with the young children's lack of experience with some of the objects, such as tools and musical instruments. The visual complexity level of the pictures for young children was higher than that for the older children, which can be explained by the fact that younger children have lower working memory capacity for processing some of the pictures. Previous researches have indicated that almost all measures of working memory showed a steady increase from the preschool years through to adolescence [38], [39]. In summary, results of the current study indicate that pictures possess different properties for young children and older children in their name agreement, ratings of picture familiarity and visual complexity. Comparison between our norms of Chinese children and the existing norms of Chinese adults [26] also showed similar results. Therefore, valid conclusions can only be made if age-appropriate stimuli are used in research study related to picture processing and naming. For example, a study on development of working memory updating using pictures with young and older children should carefully choose their stimuli in a way that those pictures are of similar properties (e.g., familiarity and visual complexity) for young and older children. Otherwise their conclusion on development of working memory updating might be disturbed by the differences in picture features for young and older children.

Norms for the same set of Snodgrass and Vanderwart pictures [2] have been collected in other cultures/languages with young children and such norms have been widely used in psychological research. The correlation pattern of the variables collected in the present study were consistent with children's norms in other languages (American: [30]; French: [32]) and Chinese adults' norm [26]. However, significant differences were found between Chinese and American children in familiarity, complexity and H statistic, and also between Chinese and French children on name agreement based on Expected Name. These results may reflect the cultural language differences and indicate that there are cross-cultural differences in picture properties for children from different cultures. Therefore, culturally specific norms are necessary when pictorial stimuli are involved to test children.

With this norm standardized for Chinese children, researchers will be better able to make clear conclusions regarding children's cognitive functions and reduce the influence of the picture materials themselves. The present norm is expected to be a useful and invaluable instrument for research with Chinese preschool children and might offer an important contribution to the community tool bag for psycholinguistic and neurolinguistic research.

Finally, we should mention the limitations of Snodgrass and Vanderwart picture set [2] with its black-and-white line drawings which showed neither surface detail nor color. Both surface detail and color have been demonstrated to improve naming performances of healthy adults (but not of Alzheimer patients) and they are more beneficial to the naming of living than nonliving things [40]. Especially color information was proposed to be crucial for recognizing objects when shape alone is inadequate for disambiguating the object [27], [41]. A recent review and meta-analysis revealed that color diagnosticity is of greatest moderator effect on the influence of color in object recognition, and that color is particularly important for recognizing nonliving things or line drawings [42]. In the current study we chose Snodgrass and Vanderwart [2]corpus to set up the first picture norm for Chinese children, mainly because it has previously built norms for children in other languages and also for Chinese adults, which makes cross-age and cross-culture comparison possible. Based on the current norm, further Chinese norms on picture sets with surface detail and color information should be built on available colorized picture databases [27], [43] and color photographs [44].

## Supporting Information

Appendix S1 260 Picture Norm for Chinese K1 and K3 Children.
(XLSX)

Appendix S2 Alternative Names Given by K1 and K3 Children for 260 pictures.
(XLSX)

Table S1 Yoon Modal Names Replaced by New Names as Expected Names
(DOCX)

Table S2 Summary Statistics of Variables for Young Children (K1) and Older Children (K3)
(DOCX)

Table S3 Numbers of Modal Names Classified into Different Categories
(DOCX)

Table S4 Correlations Among the Variables collected in this study
(DOCX)

Table S5 Correlation of Variables in the Present Norm with the Variables in Chinese Adult Norms
(DOCX)

Table S6 Correlation of Variables in the Present Norm with the Variables in American and French Children Norms
(DOCX)
