# Supplementary material for: Picture Norms for Chinese Preschool Children: Name Agreement, Familiarity, and Visual Complexity
Source: PLoS One. 2014 Mar 5;9(3):e90450. doi: 10.1371/journal.pone.0090450 (PMC3944013; doi:10.1371/journal.pone.0090450)
Supplement: Table S1 — Yoon Modal Names Replaced by New Names as Expected Names (DOCX) [file pone.0090450.s003.docx]

| Table S1. Yoon Modal Names Replaced by New Names as Expected Names | | | |
| --- | --- | --- | --- |
| **Picture No.** | **Intended name** | **Yoon modal name** | **New modal name** |
| 28 | bird | 麻雀 sparrow | 鸟 bird |
| 55 | chicken | 母鸡 hen | 鸡 chicken |
| 72 | desk | 写字台 writing desk | 桌子 desk |
| 88 | finger | 食指 index finger | 手指 finger |
| 167 | pen | 圆珠笔、圆球笔 ballpoint pen | 钢笔 pen |
| 170 | pepper | 南瓜 pumpkin | 青椒 green pepper |
| 175 | pitcher | 杯、杯子 cup | 水壶 water jug |
| 190 | rolling pin | 轮、轮子 wheel | 擀面杖 rolling pin |
| 223 | swan | 鹅 goose | 天鹅 swan |
| 234 | toaster | 烤箱 oven | 面包机 toaster |
| *Note*. Intended name from original Snodgrass and Vanderwart [2]. | | | |
| Yoon modal name from Yoon et al. [9]. | | |  |
