# Supplementary material for: Picture Norms for Chinese Preschool Children: Name Agreement, Familiarity, and Visual Complexity
Source: PLoS One. 2014 Mar 5;9(3):e90450. doi: 10.1371/journal.pone.0090450 (PMC3944013; doi:10.1371/journal.pone.0090450)
Supplement: Table S2 — Summary Statistics of Variables for Young Children (K1) and Older Children (K3) (DOCX) [file pone.0090450.s004.docx]

Table S2. Summary Statistics of Variables for Young Children (K1) and Older Children (K3)

|  | **Name agreement H** | |  | **Name agreement %** | |  | **Familiarity** | |  | **Visual Complexity** | |  | **Word Length** | |  | **DK Responses** | | |  | **Alternative Names** | |  | **AoA** |  | **Frequency** |
| --- | --- | --- | --- | --- | --- | --- | --- | --- | --- | --- | --- | --- | --- | --- | --- | --- | --- | --- | --- | --- | --- | --- | --- | --- | --- |
|  | **K1** | **K3** |  | **K1** | **K3** |  | **K1** | **K3** |  | **K1** | **K3** |  | **K1** | **K3** |  | **K1** | **K3** |  | | **K1** | **K3** |  |  |  |  |
| Mean | 1.40 | 1.36 |  | 0.48 | 0.56 |  | 3.60 | 3.88 |  | 2.70 | 2.31 |  | 1.77 | 1.84 |  | 3.31 | 1.78 |  |  | 4.25 | 4.49 |  | 5.70 |  | 2.19 |
| SD | 0.98 | 1.03 |  | 0.36 | 0.35 |  | 0.69 | 0.51 |  | 0.40 | 0.49 |  | 0.54 | 0.53 |  | 4.16 | 2.56 |  |  | 3.29 | 3.95 |  | 3.06 |  | 0.68 |
| N | 32 | 34 |  | 32 | 34 |  | 32 | 34 |  | 32 | 34 |  | 32 | 34 |  | 32 | 34 |  |  | 32 | 34 |  | 66 |  | 246 |
| Median | 1.32 | 1.26 |  | 0.47 | 0.65 |  | 3.71 | 3.97 |  | 2.67 | 2.30 |  | 2.00 | 2.00 |  | 2.00 | 1.00 |  |  | 4.00 | 3.00 |  | 4.66 |  | 2.19 |
| Q1 | 0.6 | 0.45 |  | 0.13 | 0.23 |  | 2.99 | 3.56 |  | 2.42 | 1.94 |  | 1.00 | 2.00 |  | 0.00 | 0.00 |  |  | 1.00 | 1.75 |  | 2.86 |  | 1.72 |
| Q3 | 2.12 | 2.15 |  | 0.82 | 0.91 |  | 4.13 | 4.25 |  | 3.03 | 2.70 |  | 2.00 | 2.00 |  | 5.00 | 2.00 |  |  | 6.00 | 6.00 |  | 8.77 |  | 2.61 |
| IQR | 1.52 | 1.71 |  | 0.70 | 0.68 |  | 1.14 | 0.69 |  | 0.62 | 0.76 |  | 1.00 | 0.00 |  | 5.00 | 2.00 |  |  | 5.00 | 4.25 |  | 5.91 |  | 0.89 |
| Min. | 0 | 0 |  | 0 | 0 |  | 2.06 | 2.44 |  | 1.63 | 1.35 |  | 1.00 | 1.00 |  | 0.00 | 0.00 |  |  | 0.00 | 0.00 |  | 1.94 |  | 0.30 |
| Max. | 3.79 | 4.21 |  | 1.00 | 1.00 |  | 4.82 | 4.75 |  | 3.77 | 3.48 |  | 3.00 | 3.00 |  | 18.00 | 15.00 |  |  | 14.00 | 20.00 |  | 11.00 |  | 3.67 |
| Skew | 0.30 | 0.55 |  | 0.04 | -0.27 |  | -0.37 | -0.66 |  | -0.05 | 0.03 |  | -0.10 | -0.14 |  | 1.54 | 2.01 |  |  | 0.68 | 1.28 |  | 0.60 |  | -0.01 |

*Note*. Name agreement %, percentage based on the expected name; word length, length in characters of the modal name; DK responses, number of DKN and DKO responses;

AoA, objective age of acquisition taken from Liu et al. [26]; frequency, word frequency taken from Cai and Brysbaert [36].

Q1, 25th percentile; Q3, 75th percentile; IQR, interquartile range; skew, (Q3-median)/(median-Q1).
