# Supplementary material for: Picture Norms for Chinese Preschool Children: Name Agreement, Familiarity, and Visual Complexity
Source: PLoS One. 2014 Mar 5;9(3):e90450. doi: 10.1371/journal.pone.0090450 (PMC3944013; doi:10.1371/journal.pone.0090450)
Supplement: Table S3 — Numbers of Modal Names Classified into Different Categories (DOCX) [file pone.0090450.s005.docx]

Table S3. Numbers of Modal Names Classified into Different Categories

| **Category** | **K1** | **K3** |
| --- | --- | --- |
| expected name | 172 | 187 |
| synonym | 8 | 12 |
| superordinate | 46 | 32 |
| subordinate | 2 | 2 |
| component | 2 | 4 |
| coordinate | 15 | 13 |
| failure | 15 | 10 |
| Total | 88 | 73 |

*Note*. K1, children from kindergarten first year;

K3, children from kindergarten third year.
