# Supplementary material for: Picture Norms for Chinese Preschool Children: Name Agreement, Familiarity, and Visual Complexity
Source: PLoS One. 2014 Mar 5;9(3):e90450. doi: 10.1371/journal.pone.0090450 (PMC3944013; doi:10.1371/journal.pone.0090450)
Supplement: Table S4 — Correlations Among the Variables collected in this study (DOCX) [file pone.0090450.s006.docx]

Table S4. Correlations Among the Variables collected in this study

|  | **H** | **%** | **Familiarity** | **Complexity** | **Length** | **AoA** | **Frequency** |
| --- | --- | --- | --- | --- | --- | --- | --- |
| **H** | — | -0.76** | -.33** | .14** | .05 | .66** | -.40** |
| **%** |  | — | .25** | -.19** | .16** | -.67** | .39** |
| **Familiarity** |  |  | — | -.54** | 0.08 | -.28** | .33** |
| **Complexity** |  |  |  | — | -0.03 | .13** | -.16** |
| **Length** |  |  |  |  | — | -0.03 | -0.23** |
| **AoA** |  |  |  |  |  | — | -.36** |
| **Frequency** |  |  |  |  |  |  | — |

*Note*. H, name agreement H; %, name agreement based on the expected name; length, word length of the modal name; AOA, objective age of acquisition taken from Liu et al. [26]; frequency, word frequency taken from Cai and Brysbaert [36].

* p < .05; ** p < .01.
