# Supplementary material for: Picture Norms for Chinese Preschool Children: Name Agreement, Familiarity, and Visual Complexity
Source: PLoS One. 2014 Mar 5;9(3):e90450. doi: 10.1371/journal.pone.0090450 (PMC3944013; doi:10.1371/journal.pone.0090450)
Supplement: Table S5 — Correlation of Variables in the Present Norm with the Variables in Chinese Adult Norms (DOCX) [file pone.0090450.s007.docx]

Table S5. Correlation of Variables in the Present Norm with the Variables in Chinese Adult Norms

|  | **K1 vs. AD in Liu** | **K3 vs. AD in Liu** |
| --- | --- | --- |
| **H** | .438** | .588** |
| **Familiarity** | .462** | .429** |
| **Complexity** | .720** | .793** |

*Note*. K1, children from kindergarten first year in the present study; K3, children from kindergarten third year in the present study; AD in Liu, Adults in Liu et al. [26]. H, name agreement H.

** *p* < .01.
