# Supplementary material for: Picture Norms for Chinese Preschool Children: Name Agreement, Familiarity, and Visual Complexity
Source: PLoS One. 2014 Mar 5;9(3):e90450. doi: 10.1371/journal.pone.0090450 (PMC3944013; doi:10.1371/journal.pone.0090450)
Supplement: Table S6 — Correlation of Variables in the Present Norm with the Variables in American and French Children Norms (DOCX) [file pone.0090450.s008.docx]

Table S6. Correlation of Variables in the Present Norm with the Variables in American and French Children Norms

|  | **K1+K3 vs. Cycowicz** | **K1 vs. Cannard age 4** | **K3 vs. Cannard age 6** |
| --- | --- | --- | --- |
| **H** | .515** | .635** | .374** |
| **%** | — | .509** | .516** |
| **Familiarity** | .371** | — | — |
| **Complexity** | .830** | — | — |
| **DKO** | .609** | — | — |
| **DKN** | .459** | — | — |

*Note*. K1, children from kindergarten first year in the present study; K3, children from kindergarten third year in the present study; Cycowicz, American children in Cycowicz et al. [30]; Cannard age 4, 4-year old French children in Cannard et al. [32]; Cannard age 6, 6-year old French children in Cannard et al. [32].

H, name agreement H; %, name agreement based on the expected name; DKO, DKN, number of “don’t know object” and “don’t know name” responses.

** *p* < .01.
